# Scanner-agnostic MRI harmonization via SSIM-guided disentanglement

**DOI:** 10.3389/frai.2026.1813948

**Published:** 2026-08-10

**Authors:** Luca Caldera, Lara Cavinato, Francesca Ieva, the Alzheimer's Disease Neuroimaging Initiative

**Affiliations:** 1 MOX Laboratory, Department of Mathematics, Politecnico di Milano, Milan, Italy; 2 Health Data Science Centre, Human Technopole, Milan, Italy

**Keywords:** Alzheimer's disease classification, brain age prediction, disentanglement, I2I translation, image harmonization, magnetic resonance imaging, SSIM-based loss

## Abstract

**Introduction:**

The variability introduced by differences in MRI scanner models, acquisition protocols, and imaging sites hinders consistent analysis and generalizability across multicenter studies.

**Methods:**

We present a novel image-based harmonization framework for 3D T1-weighted brain MRI, which disentangles anatomical content from scanner- and site-specific variations. The model incorporates a differentiable loss based on the Structural Similarity Index Measure (SSIM) to preserve biologically meaningful features while reducing inter-site variability. This formulation allows luminance, contrast, and structural components to be modeled separately during optimization. Training and validation were performed on multiple publicly available datasets spanning diverse scanners and sites, with testing on both healthy individuals and populations with pathological conditions. The proposed approach was evaluated across multiple target settings, including scanner-site-specific targets and a style-agnostic target, and compared with representative image-based harmonization benchmark methods.

**Results:**

Across these target settings, harmonization produced consistent and high-quality outputs. Visual comparisons, voxel intensity distributions, and SSIM-based metrics demonstrated that harmonized images achieved improved alignment across acquisition settings while preserving anatomical fidelity. In the style-agnostic setting, within-subject original–harmonized comparisons showed high anatomical preservation, with the structural component of SSIM reaching 0.975 ± 0.007. Appearance consistency also improved, with Wasserstein distances between mean voxel intensity distributions decreasing from 8.45 ± 5.35 before harmonization to 1.77 ± 0.62, and luminance similarity increasing from 0.952 ± 0.037 to 0.982 ± 0.017. Downstream analyses further confirmed the effectiveness of the proposed approach. For brain age prediction, mean absolute error decreased from 4.08 ± 1.16 to 2.81 ± 0.55 years following style-agnostic harmonization. For Alzheimer's disease classification, the area under the ROC curve improved from 0.857 ± 0.038 to 0.899 ± 0.024. Compared with the considered benchmark methods, the proposed framework showed stronger image-level harmonization and more consistent downstream improvements under the adopted evaluation protocol.

**Discussion:**

Overall, the proposed framework enhances cross-site image consistency, preserves anatomically relevant information, and improves downstream predictive performance, providing a robust and generalizable solution for large-scale multicenter neuroimaging studies.

## 1 Introduction

The growing availability of brain MRI datasets presents valuable opportunities for understanding neurological diseases and supporting clinical applications. However, differences in imaging protocols, scanner models, and acquisition settings introduce inconsistencies that compromise the reliability of imaging biomarkers. Scanner type and acquisition site strongly influence image characteristics such as contrast, brightness, and spatial resolution. Even identical scanner models can yield varying results due to differences in hardware, software, and maintenance, complicating multicenter studies and reducing reproducibility (Takao et al., 2011; Shinohara et al., 2017). Therefore, harmonizing MRI data across scanners and sites is essential for consistent, comparable analyses.

Harmonization techniques fall into two categories: feature-based and image-based. Feature-based methods adjust derived metrics—such as cortical thickness, regional volumes, or diffusion metrics—without altering raw images (Torbati et al., 2021; Radua et al., 2020; Moyer et al., 2020). Although computationally efficient, these methods depend heavily on preprocessing pipelines and risk propagating site-specific biases, which limit their ability to generalize across scanners and capture fine-grained voxel-level variations (Abbasi et al., 2024).

To overcome these limitations, image-based methods leverage machine learning to directly correct scanner- and site-induced variability at the voxel level. These approaches include Transformer models, Image-to-Image (I2I) translation, and Style Transfer approaches. Deep learning methods like CALAMITI (Zuo et al., 2021), MURD (Liu and Yap, 2024), IGUANe (Roca et al., 2025), STGAN (Liu et al., 2023), and DISARM (Caldera et al., 2025a,b) have shown promise in reducing site-related variability and improving harmonization for multicenter MRI studies. Although image-based methods directly address voxel-level variability, they often struggle to balance structural fidelity and visual consistency. Some approaches overemphasize appearance similarity, potentially altering anatomical content, while others preserve structure too rigidly, limiting the extent of harmonization. In addition, many models operate on 2D slices or small patches, which constrains their ability to capture global 3D context.

In this study, we propose a novel 3D MRI harmonization framework that extends the AGUIT domain translation network (Li et al., 2019)—originally designed for 2D natural images—to volumetric medical data. This adaptation enables the disentanglement of anatomical structure from scanner- and site-specific variations, allowing the generation of harmonized images that preserve biologically meaningful anatomy while substantially reducing variability across imaging sites. The main contributions of this work are threefold: (1) we design a new harmonization model tailored for medical imaging; (2) we propose a differentiable loss function based on the Structural Similarity Index (SSIM) (Wang et al., 2004), designed to explicitly exploit its separable luminance, contrast, and structural components, allowing for end-to-end optimization that enhances perceptual consistency, preserves anatomical fidelity, and promotes appearance consistency across harmonized images; and (3) we conduct extensive validation on multiple publicly available datasets encompassing diverse scanners, sites, and populations. Validation and testing included both healthy individuals and cohorts with pathological conditions, such as Autism Spectrum Disorder (ASD) and Alzheimer's Disease (AD) groups. In total, the study comprised 2,824 3D T1-weighted MRI volumes acquired from 113 imaging sites across 23 scanner-model categories, including two manufacturer-specific unknown-model categories, from the three main manufacturers, Siemens, Philips, and GE. Of these, 2,169 volumes were used for independent evaluation across healthy controls and cohorts with pathological conditions. This evaluation setting allowed us to assess the proposed framework under heterogeneous acquisition conditions, including scanner models and manufacturers not represented during training.

The remainder of this paper is organized as follows: Section 2 describes the proposed harmonization framework; Section 3 outlines the datasets and image preprocessing steps; Section 4 details the experimental setup; Section 5 reports and discusses the experimental results; and Section 6 concludes the paper and outlines future directions.

## 2 Methodology

This section presents the proposed harmonization framework. We first introduce the mathematical formulation of the problem, followed by a detailed description of the model architecture and the training procedure.

### 2.1 Mathematical formulation

Let *x* ∈ ℝ^1 × *H*×*W*×*D*^ denote a 3D MR image, and let $X_{u}$ and $X_{l}$ denote the sets of unlabeled and labeled images, respectively. Each labeled image in $X_{l}$ is associated with a label vector $l\in L={\left\{-1,1\right\}}^{K}$, where *K* is the number of scanners and sites considered. Importantly, $X_{u}$ and $X_{l}$ do not represent different imaging domains, but rather the same type of MR images used with or without scanner-site label annotations in the semi-supervised training setting. In each label vector, the entry corresponding to the scanner and site where the image was acquired is set to 1, with all other entries set to –1. We assume that each image can be disentangled into two latent spaces:

- the anatomical space B, which encodes the structural information of the brain, and
- the style space S, which captures the non-structural features of the MR scan.

Formally, an image x∈X can be represented by a pair of latent codes, where b∈B and s∈S. The style code *s* may itself be decomposed into a noise component n∈N and a label component l∈L, such that *s* = (*n, l*), where the label component *l* captures scanner- and site-specific variations, while the noise component *n* accounts for subject-specific, non-anatomical variations that introduce residual inconsistencies in MRI appearance possibly beyond scanner- or site-related effects. For clarity, key notation used in the training framework is summarized in Supplementary Table S1.

### 2.2 Model architecture

The network architecture comprises two encoders and a generator. The brain structure encoder, $E_{b}:X\to B$, maps an input image x∈X to a lower-dimensional space B, capturing anatomical features. The style encoder, $E_{s}:X\to S$, extracts non-anatomical features from the same image *x*, encoding them into a separate latent space S. The complete style vector s∈S=(N,L) is constructed by combining the noise component with the label component l∈L, which contains information about the scanner and the site of image acquisition. Finally, the generator, G:(B,S)→X, synthesizes a new image $\hat{x}\in X$ that retains the anatomical structure from B while incorporating the scanner-site style attributes from S. To enforce realistic image synthesis and disentanglement between anatomical and scanner-site representations, the framework further employs an image discriminator *D*^*x*^ and a latent anatomical discriminator *D*^*b*^, each jointly optimized for adversarial discrimination and scanner-site prediction.

#### 2.2.1 Architectural details

The proposed framework is implemented as a fully 3D convolutional image-to-image translation architecture composed of an anatomical encoder *E*_*b*_, a style encoder *E*_*s*_, a generator *G*, an image adversarial discriminator $D_{a}^{x}$, an image predictor $D_{p}^{x}$, a latent anatomical adversarial discriminator $D_{a}^{b}$, and a latent anatomical predictor $D_{p}^{b}$. All operations are fully three-dimensional, enabling the framework to exploit volumetric contextual information rather than processing slices independently.

Both encoders process single-channel volumetric MR inputs using 3D convolutional blocks with instance normalization and nonlinear activation layers. The anatomical encoder *E*_*b*_ extracts anatomical representations through 3D convolutional downsampling and residual attention blocks. The style encoder *E*_*s*_ produces a compact latent style embedding composed of a stochastic latent component of dimension *d*_*n*_ and a scanner-site attribute component of dimension *K*. The generator *G* reconstructs and translates MR volumes by combining anatomical and style representations through adaptive instance normalization (AdaIN)-based modulation.

Adversarial learning is enforced at both image and latent levels. At the image level, the multi-scale 3D discriminator *D*^*x*^ operates at multiple spatial resolutions and includes an adversarial head $D_{a}^{x}$, which distinguishes real from translated MR volumes, and a prediction head $D_{p}^{x}$, which promotes scanner-site consistency in generated images. At the latent level, *D*^*b*^ operates directly on anatomical representations and includes an adversarial head $D_{a}^{b}$, which encourages scanner-site-invariant anatomical encoding, and a prediction head $D_{p}^{b}$, which is trained to recover scanner-site information from the anatomical latent space, thereby supporting disentanglement through adversarial optimization.

Detailed architectural specifications and implementation settings are provided in Supplementary Section 2.

### 2.3 Training

**Figure 1:**
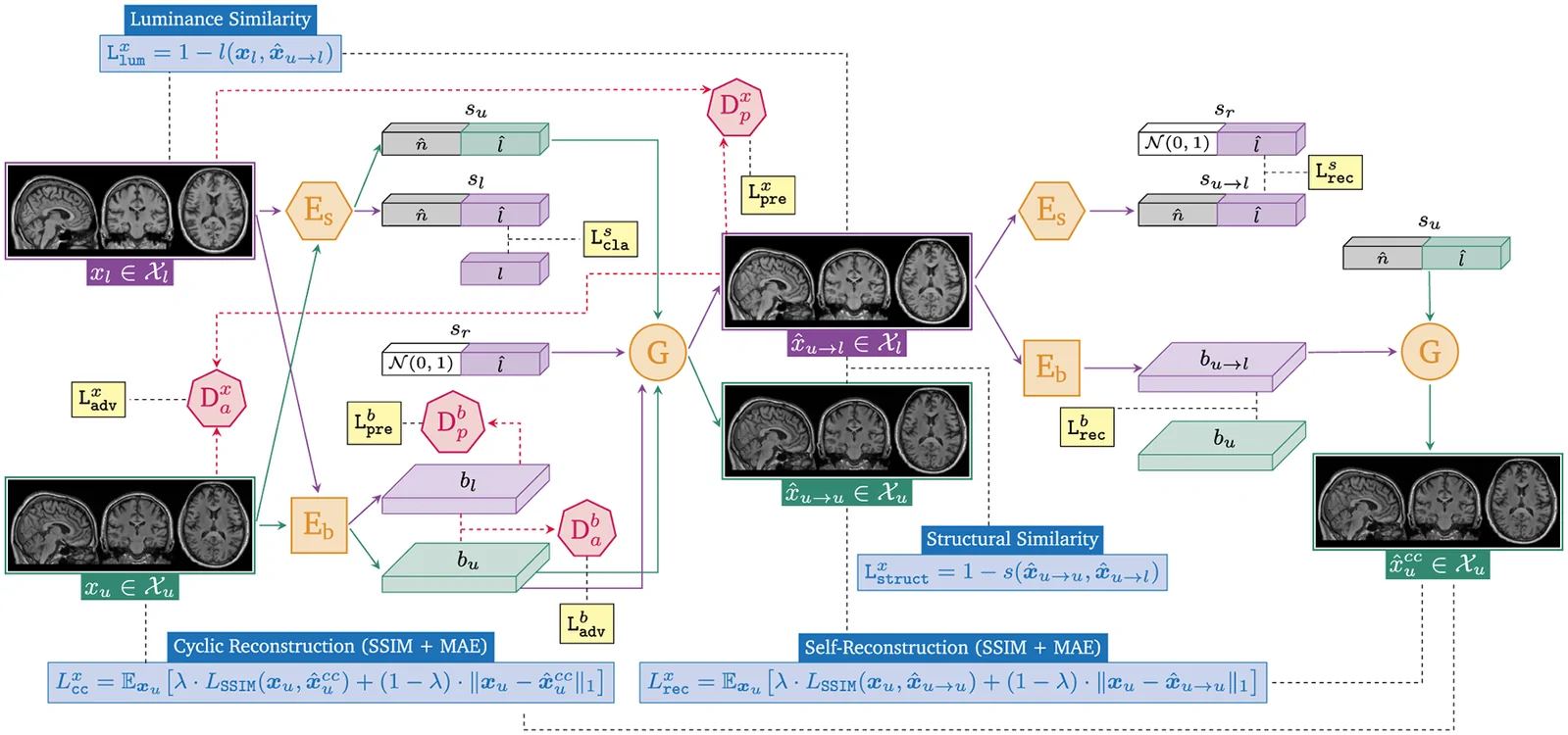
A high-level functional diagram of the model training procedure.

The training process, illustrated in Figure 1, is performed on pairs of unlabeled and labeled images, denoted as $x_{u}\in X_{u}$ and $x_{l}\in X_{l}$, respectively. Each image is independently processed by the encoders, *E*_*b*_ and *E*_*s*_, to extract latent representations: the style codes *s*_*u*_ and *s*_*l*_, and the anatomical structure codes *b*_*u*_ and *b*_*l*_. The image *x*_*u*_ is reconstructed by passing its brain structure code *b*_*u*_ and style code *s*_*u*_ to the generator. To translate scanner-site-specific characteristics, the generator is instead given *b*_*u*_ and a style code *s*_*r*_, where *s*_*r*_ is composed of random noise and the scanner-site label *l* from *x*_*l*_. The resulting generated images are denoted as ${\hat{x}}_{u\to u}$ and ${\hat{x}}_{u\to l}$, respectively. For cyclic reconstruction, the encoders reprocess ${\hat{x}}_{u\to l}$ to extract its style and brain structure codes. The brain structure code *b*_*u*→*l*_ is then passed into the generator along with the style vector *s*_*u*_ of *x*_*u*_. We denote the reconstructed image as ${\hat{x}}_{u}^{cc}$. The training objective involves minimizing a combination of loss functions, each guiding specific subnetworks in the architecture.

We devise a new Structural Similarity Index Measure (SSIM)-based loss formulation that allows separate evaluation of the image luminance *l*(*x, y*), contrast *c*(*x, y*), and structure *s*(*x, y*) components, as formulated in Equations 1 and 2:


$$\begin{array}{l} L_{\text{SSIM}}\left(x,y\right)=1-\text{SSIM}\left(x,y\right)=1-l{\left(x,y\right)}^{\alpha }\cdot c{\left(x,y\right)}^{\beta }\cdot s{\left(x,y\right)}^{\gamma } \end{array}$$ (1)

with


$$\begin{array}{l} l\left(x,y\right)=\frac{2\mu_{x}\mu_{y}+C_{1}}{\mu_{x}^{2}+\mu_{y}^{2}+C_{1}},\;c\left(x,y\right)=\frac{2\sigma_{x}\sigma_{y}+C_{2}}{\sigma_{x}^{2}+\sigma_{y}^{2}+C_{2}},\\ s\left(x,y\right)=\frac{\sigma_{xy}+C_{3}}{\sigma_{x}\sigma_{y}+C_{3}} \end{array}$$ (2)

where μ_*x*_, μ_*y*_ denote image means; σ_*x*_, σ_*y*_, their standard deviations; and σ_*xy*_, their covariance. Constants *C*_1_, *C*_2_, *C*_3_ stabilize computation, while exponents α, β, γ weight the SSIM components, allowing tailored loss functions that emphasize luminance, contrast, or structure. Unlike pixel-wise losses such as L1 or L2, SSIM jointly models perceptual similarity through these components, making it suitable for preserving anatomical integrity while promoting appearance consistency across harmonized images.

We thus further combine the SSIM with mean absolute error (MAE) in the Cycle Consistency Loss $L_{\text{cc}}^{x}$ (Equation 3) to ensure consistency in cyclic image translation, and in the Reconstruction Loss $L_{\text{rec}}^{x}$ (Equation 4) to ensure faithful reconstruction of the input image, with λ being a weighting factor:


$$\begin{array}{l} L_{\text{cc}}^{x}={\text{𝔼}}_{x_{u}}[\text{λ}\cdot L_{\text{SSIM}}\left(x_{u},{\hat{x}}_{u}^{cc}\right)+\left(1-\text{λ}\right)\cdot ∥x_{u}-{\hat{x}}_{u}^{cc}{∥}_{1}] \end{array}$$ (3)

$$\begin{array}{l} L_{\text{rec}}^{x}={\text{𝔼}}_{x_{u}}[\text{λ}\cdot L_{\text{SSIM}}\left(x_{u},{\hat{x}}_{u\to u}\right)+\left(1-\text{λ}\right)\cdot ∥x_{u}-{\hat{x}}_{u\to u}{∥}_{1}] \end{array}$$ (4)

To enforce the preservation of anatomical integrity and luminance during translation, we also introduce a Structural Consistency Loss $L_{\text{struct}}^{x}$ and a Luminance Consistency Loss $L_{\text{lum}}^{x}$, as defined in Equation 5:


$$\begin{array}{r} L_{\text{struct}}^{x}=E_{x_{u},x_{l}}[1-s\left({\hat{x}}_{u\to u},{\hat{x}}_{u\to l}\right)];\\ L_{\text{lum}}^{x}=E_{x_{u},x_{l}}[1-l\left(x_{l},{\hat{x}}_{u\to l}\right)] \end{array}$$ (5)

In addition to the proposed SSIM-based objectives, we adapt several loss components from the AGUIT framework (Li et al., 2019), reformulated for the MRI harmonization setting while preserving the disentanglement strategy introduced in AGUIT (see Figure 1).

Specifically, the brain structure discriminator $D_{a}^{b}$ receives the anatomical representations *b*_*l*_ and *b*_*u*_ from labeled and unlabeled images and is trained through the Brain Structure Adversarial Loss $L_{\text{adv}}^{b}$ to distinguish between them, while the anatomical encoder *E*_*b*_ learns a latent representation independent of scanner- and acquisition-site-specific information. The predictor $D_{p}^{b}$ attempts to recover scanner- and acquisition-site labels from the anatomical representation via the Content-Style Separation Loss $L_{\text{pre}}^{b}$, whereas *E*_*b*_ is optimized to suppress such information and enforce disentanglement between anatomical structure and scanner-site style representations.

The Style Continuity Loss $L_{\text{cla}}^{s}$ further constrains the style representation to preserve scanner- and acquisition-site attribute consistency in latent space. At the image level, the image discriminator $D_{a}^{x}$ is trained with the Image Adversarial Loss $L_{\text{adv}}^{x}$ to distinguish real images *x*_*u*_ from translated images ${\hat{x}}_{u\to l}$, encouraging the generator to produce realistic harmonized outputs while preserving anatomical content.

In parallel, the predictor $D_{p}^{x}$ is optimized through the Image Classification Loss $L_{\text{pre}}^{x}$ to predict scanner- and acquisition-site labels from generated images, enforcing alignment with the target scanner-site distribution. Finally, the Feature Consistency Loss *L*_lat_ regularizes the latent representations by enforcing consistency between the original and translated anatomical and style codes.

The overall objectives optimized for *E*_*s*_, *E*_*b*_, and *G*, and for $D_{a}^{b}$, $D_{a}^{x}$, $D_{p}^{b}$, and $D_{p}^{x}$, are given in Equations 6 and 7, respectively:


$$\begin{array}{l} {\text{L}}_{G,E_{b},E_{s}}={\text{λ}}_{\text{lum}}^{x}{\text{L}}_{\text{lum}}^{x}+{\text{λ}}_{\text{struct}}^{x}{\text{L}}_{\text{struct}}^{x}+{\text{λ}}_{\text{cla}}^{s}{\text{L}}_{\text{cla}}^{s}+{\text{λ}}_{\text{adv}}^{b}{\text{L}}_{\text{adv}}^{b}-\\ \;{\text{λ}}_{\text{pre}}^{b}{\text{L}}_{\text{pre}}^{b}+{\text{λ}}_{\text{rec}}^{x}{\text{L}}_{\text{rec}}^{x} \end{array}$$ (6)

$$\begin{array}{l} \;+{\text{λ}}_{\text{adv}}^{x}{\text{L}}_{\text{adv}}^{x}+{\text{λ}}_{\text{pre}}^{x,G}{\text{L}}_{\text{pre}}^{x,G}+{\text{λ}}_{\text{cc}}^{x}{\text{L}}_{\text{cc}}^{x}+{\text{λ}}_{\text{lat}}{\text{L}}_{\text{lat}}\\ {\text{L}}_{D}=-{\text{λ}}_{\text{adv}}^{b}{\text{L}}_{\text{adv}}^{b}+{\text{λ}}_{\text{pre}}^{b}{\text{L}}_{\text{pre}}^{b}-{\text{λ}}_{\text{adv}}^{x}{\text{L}}_{\text{adv}}^{x}+{\text{λ}}_{\text{pre}}^{x,D}{\text{L}}_{\text{pre}}^{x,D} \end{array}$$ (7)

### 2.4 Inference

**Figure 2:**
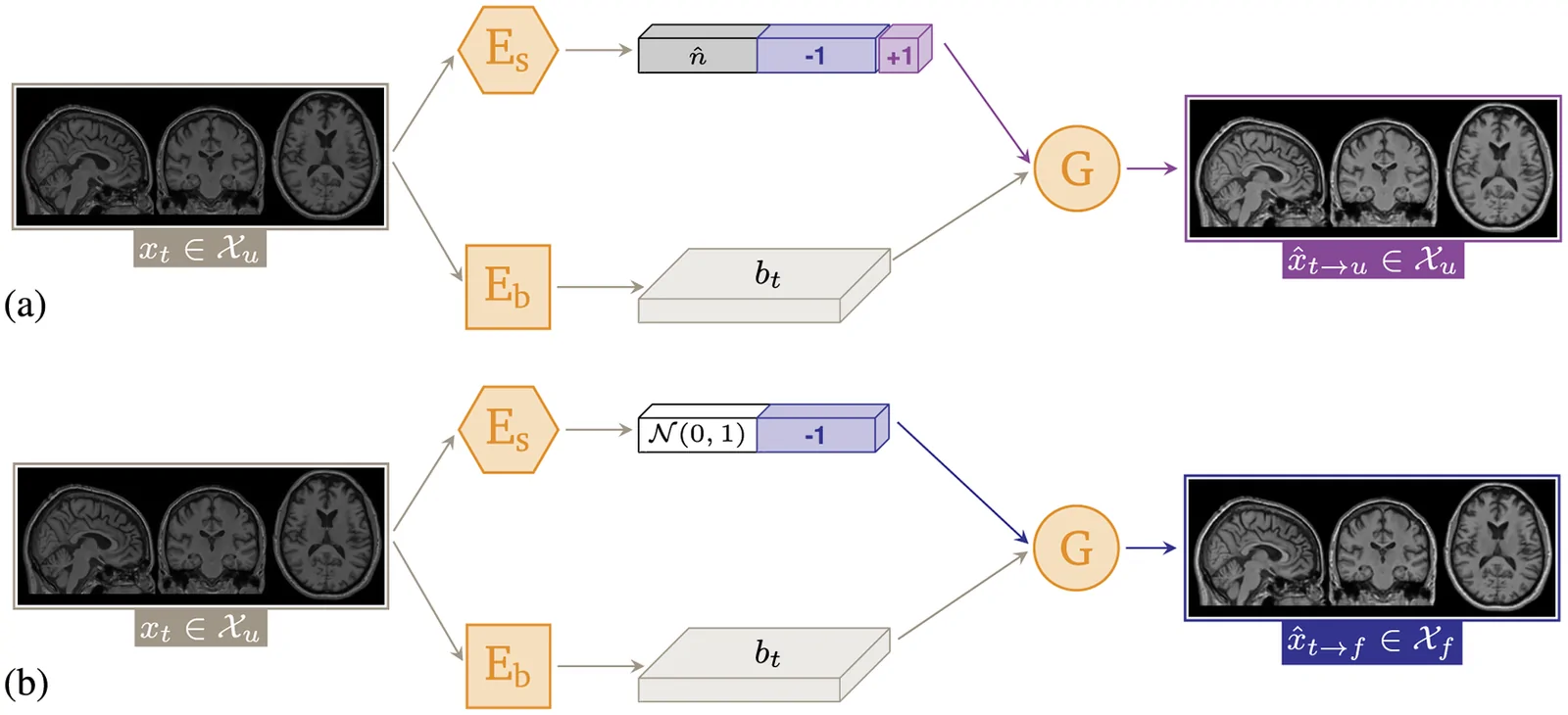
Visualizations of image generation during inference. The style code is *s* = (*n, l*), where n~N(0,1) denotes the stochastic nuisance component and *l* is the scanner-site attribute vector encoded with −1/+1 values. In **(a)**, $\hat{n}$ denotes a nuisance code extracted from a reference training image of the selected target domain. **(a)** Harmonization toward a selected target scanner-site domain learned during training, with *l* activating the target domain and the nuisance component either sampled as *n* or provided as $\hat{n}$. **(b)** Style-agnostic harmonization obtained by setting all entries of *l* to −1, so that no scanner-site attribute is activated.

At inference, a new image $x_{t}\in X$ is encoded by the anatomical encoder *E*_*b*_ to extract its anatomical representation *b*_*t*_. The harmonized image is then generated by combining *b*_*t*_ with a prescribed style code *s* = (*n, l*), where *n* denotes the noise component, interpreted as a stochastic nuisance component controlling residual non-anatomical appearance variability, and *l* denotes the scanner-site attribute vector. The noise component is either sampled from N(0,1) or, in the target-domain setting, replaced by a reference noise code $\hat{n}$ extracted from a training image belonging to the selected domain. By setting *l*, the proposed framework supports two harmonization strategies, illustrated in Figure 2.

In the first strategy, the image is harmonized toward a target scanner-site domain learned during training. The target attribute vector *l* corresponds to one of the scanner-site configurations observed in the training set. In this case, the entries of *l* associated with the desired target domain are set to +1, while the remaining entries are set to −1. The noise component can either be sampled from N(0,1) or replaced by a reference noise code $\hat{n}$ extracted from a training image belonging to the selected target domain. The former option samples residual low-level variability around the selected scanner-site conditioning, whereas the latter anchors this residual variability to a specific reference example. The generated image preserves the anatomical content of *x*_*t*_, encoded in *b*_*t*_, while exhibiting the low-level appearance characteristics associated with the selected target scanner-site domain, as shown in Figure 2a.

In the second strategy, we introduce a style-agnostic harmonization mode. In this setting, no scanner-site attribute is activated: all entries of *l* are set to −1, while the noise component *n* is sampled from N(0,1), optionally using a fixed seed for reproducibility. This configuration does not correspond to a new physical scanner-site domain. Rather, it prevents the generator from being explicitly conditioned toward any speobserved scanner-site, thereby producing images with a standardized appearance designed to reduce scanner- and site-specific cues, as shown in Figure 2b.

## 3 Data acquisition and pre-processing

We conducted experiments using T1-weighted MRI data from five large-scale, publicly available datasets, comprising both healthy controls and populations with pathological conditions across multiple acquisition sites and scanner platforms. Table 1 summarizes all datasets, including scanner models, manufacturers, acquisition sites, sources, and image counts.

**Table 1:** Overview of the training and test datasets used in this study.

Data subset	Scanner model	Manufacturer	Sites (n)	Dataset	Images (n)
**Training (** ***HC*** **)**	Prisma Fit	SIEMENS	3	ADNI 3	69
	Prisma	SIEMENS	5	ADNI 3	86
	TrioTim	SIEMENS	2	SALD, PPMI	190
	Gyroscan Intera	Philips	1	IXI	133
	Intera	Philips	1	IXI	177
**Test (** ***HC*** **)**	Unknown	GE	1	IXI	74
	Achieva dStream	Philips	3	ADNI 3, PPMI	31
	Achieva	Philips	5	ADNI 3, PPMI	27
	Skyra	SIEMENS	4	ADNI 3	38
	Gyroscan Intera	Philips	1	IXI	189
	TrioTim	SIEMENS	2	SALD, PPMI	345
**Test (** ***ASD + HC*** **)**	Signa	GE	2	ABIDE I	185
	MR750	GE	1	ABIDE I	36
	Verio	SIEMENS	2	ABIDE I	84
	Allegra	SIEMENS	3	ABIDE I	277
	Achieva	Philips	2	ABIDE I	104
	Intera	Philips	2	ABIDE I	94
	TrioTim	SIEMENS	5	ABIDE I	332
**Test (** ***AD + HC*** **)**	Prisma Fit	SIEMENS	14	ADNI 3-4	103
	Discovery MR750	GE	8	ADNI 3-4	46
	Prisma	SIEMENS	10	ADNI 3-4	46
	Skyra	SIEMENS	5	ADNI 3-4	44
	Signa Premier	GE	4	ADNI 3-4	27
	Ingenia	Philips	6	ADNI 3-4	21
	Achieva dStream	Philips	3	ADNI 3-4	13
	Verio	SIEMENS	3	ADNI 3-4	11
	Discovery MR750w	GE	3	ADNI 3-4	10
	Achieva	Philips	3	ADNI 3-4	10
	MR 7700	Philips	2	ADNI 4	7
	Signa UHP	GE	1	ADNI 4	4
	TrioTim	SIEMENS	2	ADNI 3	4
	Skyra Fit	SIEMENS	1	ADNI 4	3
	Vida	SIEMENS	1	ADNI 4	2
	Biograph mMR	SIEMENS	1	ADNI 3	1
	Unknown	Siemens	1	ADNI 3	1

The table lists scanner models, manufacturers, the number of acquisition sites, dataset sources, and image counts. Training and test sets for healthy controls (*HC*) are shown, along with the test sets containing ASD, AD, and HC participants. The “Images (n)” column indicates the number of images used.

### 3.1 Healthy control cohorts

We included data from four datasets containing healthy participants: the Alzheimer's Disease Neuroimaging Initiative (ADNI3) (Jack Jr et al., 2008), the Parkinson's Progression Markers Initiative (PPMI) (Marek et al., 2011), the IXI Brain Development Dataset (IXI) (Brain Development Project, 2019), and the Southwest University Adult Lifespan Dataset (SALD) (Wei et al., 2018).

### 3.2 Pathological cohorts

We additionally incorporated T1-weighted MR images from two large multi-site datasets containing both participants with pathological conditions and healthy controls, providing a diverse and challenging evaluation setting. The first dataset, the Autism Brain Imaging Data Exchange I (ABIDE I) (Di Martino et al., 2014), includes structural MRI data from 1,112 individuals—comprising 539 participants with autism spectrum disorder (ASD) and 573 healthy controls. ABIDE I spans 17 international sites with heterogeneous acquisition protocols, scanner manufacturers, and demographic distributions, thus offering a rigorous benchmark for evaluating the robustness and generalizability of harmonization methods across diverse scanning environments. The second dataset, the Alzheimer's Disease Neuroimaging Initiative (ADNI; Phases 3–4) (Jack Jr et al., 2008), includes participants diagnosed with Alzheimer's disease (AD) as well as healthy controls. From these phases, we included a subset of 143 participants with AD and 210 healthy controls. ADNI 3–4 provides structural MRI data from 68 imaging sites, acquired using Siemens, Philips, and GE scanners, serving as a complementary benchmark for evaluating harmonization performance in neurodegenerative populations.

### 3.3 Image pre-processing pipeline

Image pre-processing was conducted using the FSL library (Smith et al., 2004; Jenkinson et al., 2012). Initially, the images were standardized to a common orientation. Next, bias-field correction was applied to address magnetic field variations in the MRI scanner. The images were then registered to the Standard MNI152-T1-1mm space. The final pre-processed images had a shape of (1, 182, 218, 182). This common target representation provides consistent voxel spacing and matrix dimensions across datasets acquired with different native resolutions, thereby helping to reduce resolution-related variability before harmonization. Although the experiments in this study were conducted using this standardized 1 mm isotropic representation, the fully convolutional nature of the proposed architecture allows it to be adapted to other spatial resolutions or matrix sizes, provided that the pre-processing setup and any input-dependent network settings are adjusted consistently.

## 4 Experimental setup

This section describes the experimental design, evaluation criteria, and benchmark comparisons used to assess the proposed harmonization framework. Detailed architectural and implementation settings, including latent dimensions, loss weights, training configuration, computational cost, and inference runtime, are reported in Supplementary Section 2.

The model was trained on a dataset of 655 T1-weighted MR images from healthy controls, acquired across 5 scanners and 12 sites. For evaluation, we used an independent test set comprising 704 healthy control images collected using 6 scanners across 16 sites. Additional test sets included both participants with pathological conditions and healthy controls from the ABIDE I and ADNI 3–4 datasets. A detailed summary of the training and test datasets is presented in Table 1.

In accordance with the inference strategies described in Section 2.4 and illustrated in Figure 2, we evaluated the proposed method within two harmonization frameworks. First, in the target scanner-site harmonization framework, all test images were mapped to selected scanner-site domains learned during training. Specifically, we considered two distinct scanner-site pairs, referred to as A and B: Gyroscan Intera—Guy's Hospital and TrioTim—Southwest University, China. Second, in the style-agnostic harmonization framework, all scanner-site attributes were deactivated to generate images without explicitly imposing the appearance of any specific training domain. For the target scanner-site settings A and B, the nuisance component was fixed to a reference code $\hat{n}$ extracted from a representative training image belonging to the selected target domain.

The effectiveness of the harmonization was evaluated using four complementary criteria: (i) preservation of anatomical structures, (ii) consistency of image appearance across sites, (iii) performance on downstream predictive tasks, and (iv) reduction of residual scanner-site information in the harmonized images. In addition, axial, coronal, and sagittal slices were visually inspected to qualitatively assess the realism and inter-site consistency of the harmonized images.

### 4.1 Preservation of anatomical structure

We used the structural component of the SSIM to assess the preservation of anatomical structures following harmonization. Given the absence of ground-truth harmonized images, we focused on the structural component of SSIM computed between each original image and its harmonized counterpart. This metric quantifies the extent to which the underlying anatomical features are maintained after harmonization. Specifically, we computed SSIM values across image pairs representing diverse anatomical structures, as well as across the entire test set, for all three harmonization targets (scanner-site pairs A and B, and the style-agnostic target), considering each harmonized–original image pair.

### 4.2 Consistency of image appearance

We assess appearance consistency by comparing mean voxel intensity distributions across scanner-site pairs before and after harmonization. To quantify alignment, we compute the Wasserstein distance between all pairs of scanner-site distributions, both pre- and post-harmonization, for all three harmonization targets. We report the mean and standard deviation in both cases. Additionally, to further assess consistency, we calculate the SSIM, focusing on the luminance component, between all pairs of original test images and, separately, all pairs of harmonized images. Again, we report the mean and standard deviation before and after harmonization. These analyses were conducted using the test dataset composed exclusively of healthy controls (Table 1).

### 4.3 Downstream analysis

To evaluate the impact of harmonization on downstream neuroimaging applications, we performed two predictive tasks using convolutional neural networks (CNNs): (i) *age prediction* and (ii) *disease classification*. In both tasks, we employed 3D convolutional architectures based on the ResNet family (He et al., 2016). Each model was trained separately on non-harmonized and harmonized data to assess the effect of harmonization on predictive performance. Model evaluation was conducted using stratified 5-fold cross-validation, reporting the mean and standard deviation of the respective metrics. To prevent data leakage, all cross-validation splits were performed at the subject level. When repeated acquisitions were available, all scans from the same participant were assigned to the same fold. The harmonization model was trained independently and kept fixed during downstream evaluation. Test folds were never used for hyperparameter tuning, model selection, early stopping, or optimization of either the harmonization model or the downstream predictive models.

For *age prediction*, the CNN was trained to estimate chronological age from structural MRI scans using the ABIDE I dataset. Performance was assessed via mean absolute error (MAE), root mean squared error (RMSE), and the coefficient of determination (*R*^2^).

For *disease classification*, a binary 3D ResNet model was trained to distinguish Alzheimer's disease (AD) patients from healthy controls using the ADNI 3–4 dataset. Evaluation metrics included balanced accuracy, F1-score, and area under the receiver operating characteristic curve (AUC).

### 4.4 Residual scanner-site predictability

To assess whether harmonization reduced residual acquisition-domain information, we performed a scanner-site prediction analysis on the independent healthy-control test set. Scanner-site labels were defined as the combination of scanner model and site, consistently with the acquisition-domain definition used throughout the manuscript. Scanner-site classes with fewer than 10 subjects were excluded to avoid unstable estimates from small classes. After filtering, the analysis included 667 healthy-control subjects distributed across 7 scanner-site classes.

Scanner-site prediction was performed using the same 3D ResNet architecture adopted for the downstream predictive tasks, replacing the task-specific output layer with a multiclass scanner-site classification head. For each image condition, namely pre-harmonization and each harmonized target setting, a separate classifier was trained and evaluated using 5-fold cross-validation. Balanced accuracy and macro-F1 were used as evaluation metrics, as they are suitable for multiclass classification under class imbalance. The corresponding results are reported in Section 5.4.

### 4.5 Benchmark methods

To provide a comparative evaluation, we considered three image-level harmonization benchmarks: IGUANe (Roca et al., 2025), STGAN (Liu et al., 2023), and DISARM (Caldera et al., 2025a).

These methods cover complementary harmonization strategies: STGAN performs style transfer toward a reference scanner, IGUANe adopts a many-to-one adversarial framework to map images from multiple sites to a common reference domain, whereas DISARM disentangles anatomical and scanner-specific information to generate scanner-free harmonized images. IGUANe and STGAN operate on skull-stripped images, whereas the proposed model and DISARM were evaluated in the non-skull-stripped setting. Accordingly, structural preservation metrics involving skull-stripped and non-skull-stripped methods should be interpreted in light of these different preprocessing settings. Complete implementation and preprocessing details for each benchmark are reported in Supplementary Section 3.

## 5 Results

In this section, we present the results of the experiments described in Section 4.

**Figure 3:**
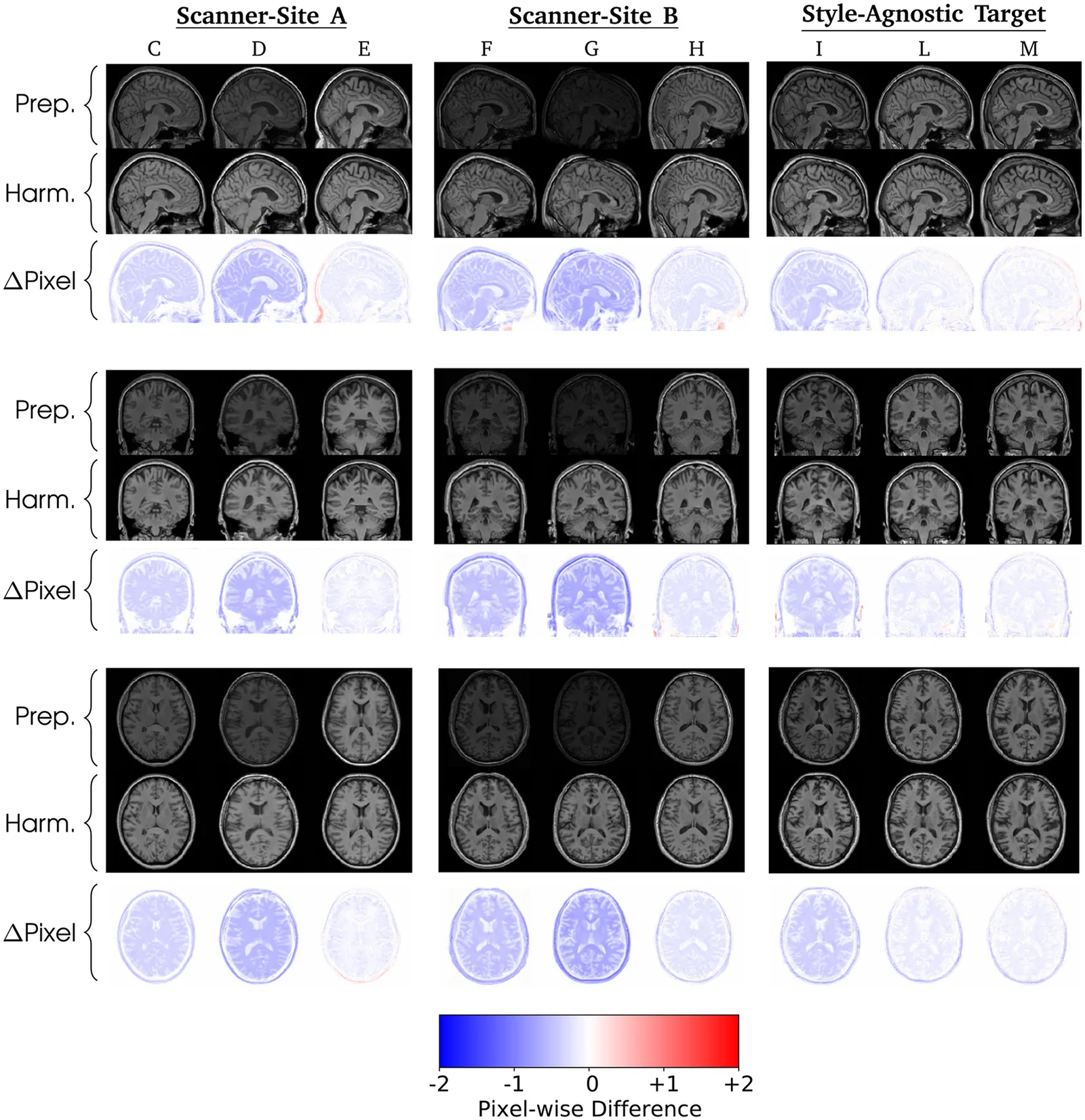
Sagittal, coronal, and axial slices from 9 MR images, both from the original scans (C-M) and their harmonized versions according to scanner-site pair A (left column), scanner-site pair B (middle column), and a style-agnostic target (right column). Images C, D, and E are taken from the HC test dataset, F, G, and H from the ABIDE dataset, and I, L, and M from the ADNI dataset. Each letter corresponds to a different scanner–site pair used for image acquisition. Heatmaps indicate pixel-wise differences between the original and harmonized images. For the same representative images shown in Figure 3, voxel-intensity histograms before and after harmonization are reported in Supplementary Figure S1, providing an additional qualitative visualization of the improved alignment of intensity distributions across scanner-site pairs.

Figure 3 provides a visual comparison of the harmonization outcomes across the three target domains. The original images exhibit substantial inter-domain variability, whereas the harmonized outputs demonstrate visually consistent appearances across all style targets. This consistency is particularly evident in the heatmaps, which illustrate pixel-wise differences. The anatomical structures remain visually well-preserved after harmonization, indicating that the proposed method effectively reduces style-related variability while maintaining underlying anatomical integrity.

Nevertheless, visual assessment alone is insufficient to draw definitive conclusions regarding the quality of harmonization or the preservation of anatomical details. Therefore, we complement the qualitative evaluation with quantitative analyses to rigorously assess both the harmonization performance and the fidelity of anatomical preservation.

### 5.1 Preservation of anatomical structure

To provide a reference for inter-subject anatomical variability, we first computed the structural SSIM across 100 randomly selected pairs of subjects, obtaining an average of 0.680 ± 0.050 for the non-skull-stripped setting used by the proposed model and DISARM. Table 2 summarizes the structural similarity preservation results for the proposed model and the benchmark methods. We then computed the same metric between each original image and its harmonized version, i.e., within-subject original–harmonized pairs. Across all test images and the three harmonization targets, the proposed model achieved consistently high structural preservation, with original–harmonized Struct-SSIM values of 0.975 ± 0.007, 0.975 ± 0.009, and 0.975 ± 0.007 for scanner-site A, scanner-site B, and the style-agnostic target, respectively.

**Table 2:** Structural similarity preservation assessed using Struct-SSIM.

Method	Inter-subject reference *s*(*x, y*)	Original vs. harmonized *s*(*x, y*)
Proposed model		
A (*scanner-site*)	0.680 ± 0.050	0.975 ± 0.007
B (*scanner-site*)	0.680 ± 0.050	0.975 ± 0.009
Style-agnostic	0.680 ± 0.050	0.975 ± 0.007
Benchmarks		
IGUANe	0.911 ± 0.022	0.998 ± 0.002
STGAN	0.904 ± 0.023	0.998 ± 0.001
DISARM	0.680 ± 0.050	0.966 ± 0.010

Inter-subject reference values were computed on randomly selected pairs of different subjects and provide a baseline for anatomical variability. For IGUANe and STGAN, reference values were computed separately because these methods operate on skull-stripped images. Original–harmonized Struct-SSIM values quantify within-subject anatomical preservation after harmonization. Values are reported as mean ± standard deviation.

For benchmark methods based on skull-stripped images, structural SSIM values were computed within their corresponding preprocessing setting. In this setting, the removal of non-brain tissues increases the proportion of homogeneous background, leading to higher inter-subject reference values: 0.911 ± 0.022 for IGUANe and 0.904 ± 0.023 for STGAN. Consequently, the very high original–harmonized Struct-SSIM values obtained by IGUANe and STGAN, both close to 0.998, should be interpreted relative to these higher reference similarities rather than directly compared with the non-skull-stripped setting. Under the same non-skull-stripped reference setting as the proposed model, DISARM achieved an original–harmonized Struct-SSIM of 0.966 ± 0.010, slightly lower than the values obtained by the proposed framework across all three target settings.

### 5.2 Consistency of image appearance

**Figure 4:**
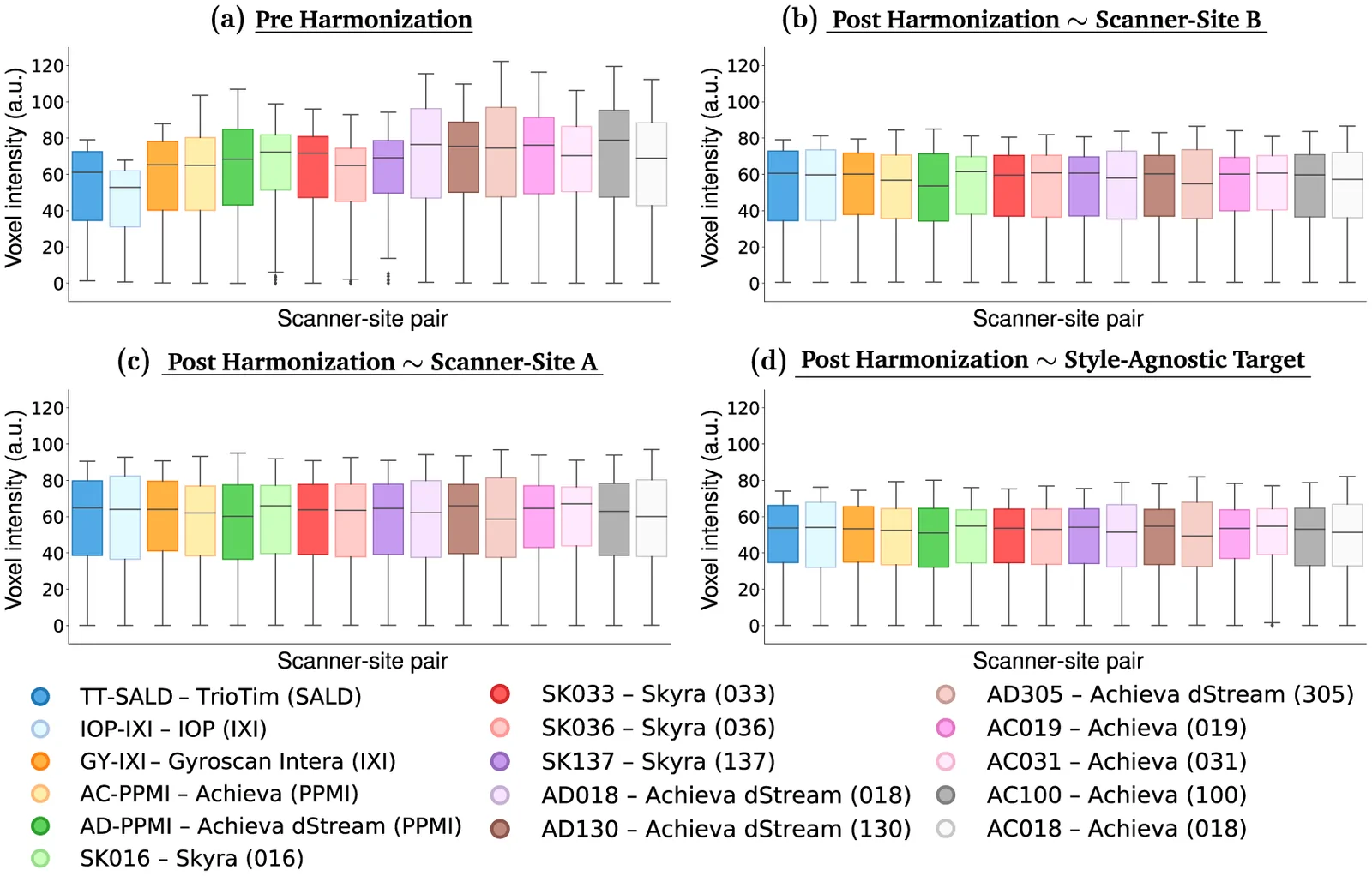
Comparison of voxel intensity mean distributions for each scanner-site pair in the test images, before **(a)** and after harmonization to scanner-site pair B **(b)**, scanner-site pair A **(c)**, and the style-agnostic target **(d)**.

We compared the voxel intensity mean distributions for each of the 16 scanner-site pairs in the test images, both before and after harmonization with respect to the three target attributes (Figure 4). As shown in the plots, the proposed model achieves strong alignment of voxel intensity distributions across all target types, consistent with visual inspection.

**Figure 5:**
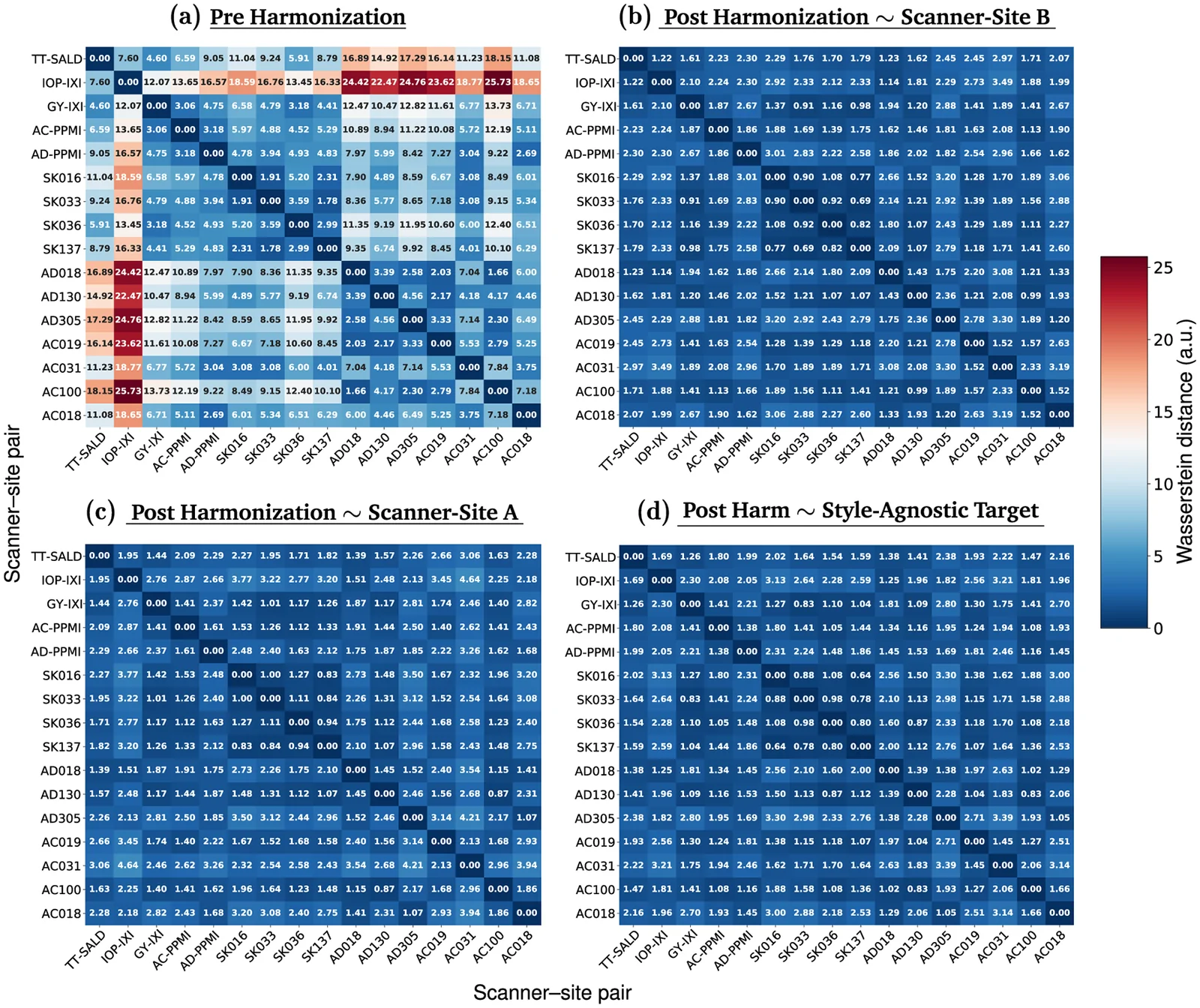
Heatmaps showing the pairwise Wasserstein distances between mean voxel intensity distributions for all scanner-site pairs in the healthy control test dataset. **(a)** shows distances before harmonization, while **(b–d)** show distances after harmonization to scanner-site pair B, scanner-site pair A, and the style-agnostic target, respectively. Row and column labels correspond to the scanner-site abbreviations defined in Figure 4.

This observation is quantitatively supported by the results summarized in Table 3. The Wasserstein distance between scanner-site distributions is markedly reduced after harmonization, from 8.454 ± 5.347 pre-harmonization to 2.083 ± 0.771, 1.912 ± 0.640, and 1.768 ± 0.624 for targets A, B, and the style-agnostic target, respectively. This substantial reduction indicates that intensity distributions across scanners and sites are brought into close agreement, effectively mitigating scanner- and site-related variability. Corresponding heatmaps in Figure 5 visualize the pairwise Wasserstein distances.

**Table 3:** Evaluation of luminance similarity between images and Wasserstein distance between the mean voxel intensity distributions.

Method	Wasserstein distance	Luminance similarity
Pre-harmonization	8.45 ± 5.35	0.95 ± 0.04
Proposed model		
A (*scanner-site*)	2.08 ± 0.77^***^	0.98 ± 0.02^***^
B (*scanner-site*)	1.91 ± 0.64^***^	0.98 ± 0.02^***^
Style-agnostic	1.77 ± 0.62^***^	0.98 ± 0.02^***^
Benchmarks		
IGUANe	4.58 ± 2.42^***^	0.97 ± 0.03^**^
STGAN	4.23 ± 2.38^***^	0.97 ± 0.03^**^
DISARM	2.80 ± 0.88^***^	0.97 ± 0.02^**^

Values are reported as mean ± standard deviation before harmonization (Pre-Harmonization), after harmonization with the proposed model under the three target settings, and after harmonization with benchmark methods. Asterisks indicate statistical significance from Wilcoxon signed-rank tests against pre-harmonization values (^*^p < 0.05, ^**^p < 0.01, ^***^p < 0.001).

Similarly, the luminance similarity increases from 0.952 ± 0.037 pre-harmonization to 0.981 ± 0.017, 0.982 ± 0.017, and 0.982 ± 0.017 after harmonization to the three targets. These results demonstrate that the harmonized images not only exhibit well-aligned voxel intensity distributions but also maintain high voxel-level luminance consistency across sites.

When compared with the benchmark methods, the proposed model achieved stronger appearance harmonization. Although all benchmark approaches reduced scanner-site variability relative to the pre-harmonization condition, their Wasserstein distances remained higher than those obtained with the proposed framework. Specifically, IGUANe, STGAN, and DISARM achieved Wasserstein distances of 4.58 ± 2.42, 4.23 ± 2.38, and 2.80 ± 0.88, respectively, whereas the proposed model reached lower values across all target settings, with the style-agnostic target yielding the lowest distance of 1.77 ± 0.62. Luminance similarity also improved for all methods, with benchmark values around 0.97, while the proposed model reached approximately 0.98 across the three harmonization targets.

### 5.3 Downstream analysis

For *age prediction*, Table 4 shows that the proposed harmonization framework consistently improved predictive performance compared with the pre-harmonization condition. The mean absolute error (MAE) decreased from 4.08 ± 1.16 years pre-harmonization to 2.84 ± 0.46, 3.00 ± 0.51, and 2.81 ± 0.55 years for scanner-site A, scanner-site B, and the style-agnostic target, respectively. Similarly, the coefficient of determination (*R*^2^) increased from 0.473 ± 0.254 to 0.710 ± 0.099, 0.688 ± 0.082, and 0.713 ± 0.122, while the root mean squared error (RMSE) decreased from 5.72 ± 1.64 to 4.26 ± 0.68, 4.45 ± 0.61, and 4.25 ± 1.13 across the three targets. These results indicate that the proposed harmonization procedure reduces acquisition-related variability while preserving age-related anatomical information relevant for regression.

**Table 4:** Performance of the age prediction model before and after data harmonization.

Method	MAE	RMSE	*R* ^2^
Pre-harmonization	4.08 ± 1.16	5.72 ± 1.64	0.473 ± 0.254
Proposed model			
A (*scanner-site*)	2.84 ± 0.46	4.26 ± 0.68	0.710 ± 0.099
B (*scanner-site*)	3.00 ± 0.51	4.45 ± 0.61	0.688 ± 0.082
Style-agnostic	2.81 ± 0.55	4.25 ± 1.13	0.713 ± 0.122
Benchmarks			
IGUANe	3.29 ± 0.56	4.98 ± 0.76	0.611 ± 0.098
STGAN	3.19 ± 0.94	4.74 ± 1.44	0.626 ± 0.251
DISARM	3.17 ± 0.40	4.44 ± 0.66	0.693 ± 0.064

Values are reported as mean ± standard deviation before harmonization, after harmonization with the proposed model under the three target settings, and after harmonization with benchmark methods. The table reports mean absolute error (MAE), root mean squared error (RMSE), and coefficient of determination (*R*^2^) across folds.

Compared with the benchmark methods, the proposed model achieved the best overall age-prediction performance. IGUANe, STGAN, and DISARM also improved over the pre-harmonization baseline, reducing MAE to 3.29 ± 0.56, 3.19 ± 0.94, and 3.17 ± 0.40, respectively. However, all three benchmark methods remained less accurate than the proposed framework, particularly compared with the style-agnostic and scanner-site A targets.

For *Alzheimer's disease (AD) classification*, harmonization also resulted in consistent performance gains for the proposed model (Table 5). The area under the ROC curve (AUC) improved from 0.857 ± 0.038 pre-harmonization to 0.896 ± 0.026, 0.897 ± 0.025, and 0.899 ± 0.024 for scanner-site A, scanner-site B, and the style-agnostic target, respectively. Balanced accuracy increased from 0.783 ± 0.046 to 0.806 ± 0.033, 0.817 ± 0.022, and 0.832 ± 0.035, while the F1-score rose from 0.744 ± 0.052 to 0.768 ± 0.040, 0.783 ± 0.026, and 0.800 ± 0.042. These improvements suggest that the harmonized images retain disease-relevant anatomical patterns while reducing scanner- and site-dependent confounding effects.

**Table 5:** Classification performance for AD versus healthy controls on MRI data before and after harmonization.

Method	AUC	Balanced accuracy	F1-score
Pre-harmonization	0.857 ± 0.038	0.783 ± 0.046	0.744 ± 0.052
Proposed model			
A (*scanner-site*)	0.896 ± 0.026	0.806 ± 0.033	0.768 ± 0.040
B (*scanner-site*)	0.897 ± 0.025	0.817 ± 0.022	0.783 ± 0.026
Style-agnostic	0.899 ± 0.024	0.832 ± 0.035	0.800 ± 0.042
Benchmarks			
IGUANe	0.857 ± 0.059	0.767 ± 0.066	0.717 ± 0.082
STGAN	0.875 ± 0.025	0.789 ± 0.048	0.743 ± 0.063
DISARM	0.884 ± 0.020	0.785 ± 0.039	0.740 ± 0.055

Values are reported as mean ± standard deviation before harmonization, after harmonization with the proposed model under the three target settings, and after harmonization with benchmark methods. Metrics robust to class imbalance are reported across folds.

The benchmark methods showed more limited and less consistent improvements in the AD classification task. IGUANe did not improve AUC relative to the pre-harmonization condition and yielded lower balanced accuracy and F1-score. STGAN and DISARM increased AUC to 0.875 ± 0.025 and 0.884 ± 0.020, respectively, but their balanced accuracy and F1-score remained below those obtained with the proposed framework. In particular, the style-agnostic target achieved the strongest classification performance overall, with the highest AUC, balanced accuracy, and F1-score among all tested harmonization settings.

### 5.4 Residual scanner-site predictability

Consistent with the evaluation protocol described in Section 4.4, we assessed whether scanner-site information could still be recovered from the images after harmonization. As shown in Table 6, scanner-site prediction from the original images was high, with a balanced accuracy of 0.921 ± 0.071 and a macro-F1 of 0.899 ± 0.092, confirming that acquisition-domain information was strongly recoverable before harmonization.

**Table 6:** Scanner-site prediction performance before and after harmonization on the independent healthy-control test set.

Method	Balanced accuracy	Macro-F1
Pre-harmonization	0.921 ± 0.071	0.899 ± 0.092
A (*scanner-site*)	0.730 ± 0.084	0.697 ± 0.107
Δ A	−0.191	−0.202
B (*scanner-site*)	0.768 ± 0.035	0.727 ± 0.046
Δ B	−0.153	−0.173
Style-agnostic	0.610 ± 0.222	0.545 ± 0.267
Δ style-agnostic	−0.311	−0.354

Values are reported as mean ± standard deviation across stratified cross-validation folds. Δ denotes harmonized minus pre-harmonization performance.

After harmonization, scanner-site prediction performance decreased across all target settings. The style-agnostic setting yielded the largest reduction, with balanced accuracy decreasing to 0.610 ± 0.222 and macro-F1 to 0.545 ± 0.267. Harmonization toward scanner-site A also substantially reduced scanner-site predictability, reaching 0.730 ± 0.084 balanced accuracy and 0.697 ± 0.107 macro-F1, while target B yielded 0.768 ± 0.035 and 0.727 ± 0.046, respectively.

These findings indicate that the proposed harmonization framework attenuates, but does not fully remove, scanner-site information from the images. This is consistent with the expected role of image harmonization, which is to reduce acquisition-related confounding while preserving biologically meaningful image content, rather than eliminating all acquisition-domain signatures. Residual predictability may reflect acquisition-domain cues associated with resolution, protocol differences, preprocessing sensitivity, or cohort composition. In the present non-skull-stripped setting, it may also partly arise from information outside the brain, including field-of-view, background, or head-coverage differences that vary systematically across datasets or acquisition sites.

Taken together with the high anatomical preservation, improved appearance consistency, and downstream performance gains reported above, these results suggest that the proposed framework reduces acquisition-domain information while preserving biologically and clinically relevant image content.

## 6 Conclusion

We presented a novel 3D T1-weighted MRI harmonization framework that disentangles subject-specific anatomical content from scanner- and site-related appearance variability. By combining reconstruction, cyclic reconstruction, adversarial objectives, and SSIM-guided structural and appearance consistency constraints, the proposed method reduces acquisition-related variability while preserving individual anatomical information. This SSIM-guided anatomy/style disentanglement enables harmonized images to retain subject-specific structural features while improving appearance alignment across acquisition domains.

Extensive evaluation across multiple large-scale datasets, including healthy controls and cohorts with pathological conditions such as ASD and AD, showed that the proposed framework produces harmonized images with improved voxel-intensity distribution alignment, increased luminance consistency, and preserved anatomical structure. The benefits of harmonization were also reflected in downstream neuroimaging analyses, where brain age prediction errors decreased after harmonization and Alzheimer's disease classification performance improved across the main evaluation metrics. In addition, residual scanner-site prediction analyses showed that harmonization reduced the recoverability of acquisition-domain information from the images. Together, these findings indicate that the proposed method attenuates acquisition-related confounding effects while retaining biologically and clinically relevant information.

Compared with the considered image-based benchmark methods, the proposed framework showed stronger distributional alignment and more consistent improvements in downstream tasks, supporting the contribution of SSIM-guided anatomy/style disentanglement to MRI harmonization.

Some aspects nevertheless warrant further investigation. Although the present evaluation provides evidence of robustness across independent datasets, unseen scanner models, an unseen manufacturer, and cohorts with pathological conditions, additional validation on larger and more diverse multicenter cohorts, including traveling-subject datasets, would further strengthen the assessment of generalizability. Future work should also examine the impact of harmonization on regional morphometric measures and task-specific neuroimaging biomarkers, such as cortical thickness, subcortical volumes, and disease-sensitive anatomical features, using validated segmentation pipelines. Finally, while all images in this study were mapped to a common 1 mm isotropic MNI space to ensure a consistent input representation, further experiments could assess the behavior of the model under alternative preprocessing pipelines, spatial resolutions, and input matrix sizes.

Overall, the proposed framework represents a promising and scalable image-based harmonization strategy for large-scale multicenter neuroimaging studies. By combining SSIM-guided anatomy/style disentanglement with scanner-site conditioning, it provides a flexible approach for reducing acquisition-related variability while preserving anatomical information and supporting downstream predictive analyses.

## Data Availability

The original contributions presented in the study are included in the article/Supplementary material, further inquiries can be directed to the corresponding author.

## Group member of the Alzheimer's Disease Neuroimaging Initiative

Data used in preparation of this article were obtained from the Alzheimer's Disease Neuroimaging Initiative (ADNI) database (adni.loni.usc.edu). As such, the investigators within ADNI contributed to the design and implementation of ADNI and/or provided data but did not participate in analysis or writing of this report. A complete listing of ADNI investigators can be found at: http://adni.loni.usc.edu/wp-content/uploads/how_to_apply/ADNI_Acknowledgement_List.pdf.
