# Supplementary material for: Scanner-agnostic MRI harmonization via SSIM-guided disentanglement
Source: Front Artif Intell. 2026 Aug 10;9:1813948. doi: 10.3389/frai.2026.1813948 (PMC13500658; doi:10.3389/frai.2026.1813948)
Supplement: Supplementary file 1 [file Supplementary_file_1.pdf]

# Supplementary Material

## 1 NOTATION

| Symbol | Description |
|---|---|
| $\boldsymbol{x}_u, \boldsymbol{x}_l$ | Unlabeled and labeled input MR images, respectively. |
| $\boldsymbol{l}$ | Scanner-site attribute vector with entries in $\{-1, 1\}$. The entry corresponding to the target scanner and site is set to $+1$, while all others are set to $-1$. |
| $E_b, E_s, G$ | Anatomical encoder, style encoder, and generator/decoder, respectively. $E_b$ extracts anatomical features, $E_s$ extracts style features, and $G$ synthesizes images from anatomical and style codes. |
| $\boldsymbol{b}_u, \boldsymbol{b}_l$ | Anatomical latent representations extracted from $\boldsymbol{x}_u$ and $\boldsymbol{x}_l$, respectively. |
| $\boldsymbol{s}_u, \boldsymbol{s}_l$ | Style latent representations extracted from $\boldsymbol{x}_u$ and $\boldsymbol{x}_l$, composed of a stochastic component $\boldsymbol{n}$ and a scanner-site label component $\boldsymbol{l}$, i.e., $\boldsymbol{s} = (\boldsymbol{n}, \boldsymbol{l})$. |
| $\boldsymbol{n}$ | Stochastic nuisance component of the style representation, modelling residual non-anatomical appearance variability. It can be extracted by the style encoder or sampled from a standard normal distribution when constructing a target style code. |
| $\boldsymbol{s}_r$ | Random target style representation obtained by combining a sampled noise vector $\boldsymbol{n}$ with the scanner-site label vector $\boldsymbol{l}$. |
| $\hat{\boldsymbol{x}}_{u\to u}$ | Self-reconstructed image obtained by decoding $(\boldsymbol{b}_u, \boldsymbol{s}_u)$. |
| $\hat{\boldsymbol{x}}_{u\to l}$ | Harmonized image obtained by decoding the anatomical representation $\boldsymbol{b}_u$ with the target style representation $\boldsymbol{s}_r$. |
| $\hat{\boldsymbol{x}}_u^{cc}$ | Cycle-reconstructed image obtained by re-encoding $\hat{\boldsymbol{x}}_{u\to l}$ and decoding it with the original style representation $\boldsymbol{s}_u$. |
| $D^x$ | Image-level discriminator operating on real and generated MR images. It includes an adversarial branch $D_a^x$ and a scanner-site prediction branch $D_p^x$. |
| $D_a^x$ | Image-level adversarial discriminator used to distinguish real and translated MR images. |
| $D_p^x$ | Image-level scanner-site predictor enforcing scanner-site consistency in generated images. |
| $D^b$ | Latent anatomical discriminator operating on anatomical latent representations. It includes an adversarial branch $D_a^b$ and a scanner-site prediction branch $D_p^b$. |
| $D_a^b$ | Latent adversarial discriminator operating on anatomical representations to encourage scanner-site-invariant anatomical encoding. |
| $D_p^b$ | Latent scanner-site predictor trained to recover scanner-site labels from anatomical representations; through adversarial optimization, it encourages scanner-site information to be suppressed from the anatomical latent space. |

**Table S1.** Notation used throughout the proposed harmonization framework and in Figure 1 of the main article.

## 2 DETAILED NETWORK ARCHITECTURE AND IMPLEMENTATION DETAILS

This section provides detailed architectural specifications and implementation details of the proposed harmonization framework, including latent dimensions, loss weights, training configuration, computational cost, and inference runtime.

**Anatomical Encoder ($E_b$).** The anatomical encoder $E_b$ receives a single-channel volumetric MR image as input and produces a volumetric anatomical latent representation. The network is composed of successive 3D convolutional blocks with reflection padding, instance normalization, and ReLU activations. Each downsampling stage halves the spatial dimensions while increasing the number of feature channels. Given an input volume of size $H \times W \times D$, after $n$ downsampling stages the latent anatomical representation has dimensions:

$$d \cdot 2^n \times \frac{H}{2^n} \times \frac{W}{2^n} \times \frac{D}{2^n},$$

where $d$ denotes the base number of feature channels. In the implementation used in this study, the anatomical encoder applies a single effective spatial downsampling stage for the anatomical representation passed to the generator, corresponding to $n = 1$. Consequently, the anatomical latent code $\boldsymbol{b}$ has spatial size $\frac{H}{2} \times \frac{W}{2} \times \frac{D}{2}$.

The latent representation is further processed through four residual blocks. Each residual block consists of two 3D convolutional layers followed by channel-attention and spatial-attention modules. Residual connections are employed to preserve anatomical consistency while enabling effective contextual feature refinement.

**Style Encoder ($E_s$).** The style encoder $E_s$ extracts scanner- and acquisition-related appearance characteristics from the input volume. The network is composed of stacked 3D convolutional layers with progressive downsampling operations. The resulting style representation is decomposed into:

- a noise component $\boldsymbol{n}$ of dimension $d_n$, and
- a scanner-site attribute component $\boldsymbol{l}$ of dimension $K$,

yielding a final style representation

$$\boldsymbol{s} = (\boldsymbol{n}, \boldsymbol{l}) \in \mathcal{N} \times \mathcal{L}$$

of dimension $d_n + K$.

**Generator ($G$).** The generator reconstructs or translates MR volumes by combining the anatomical latent representation with the style representation. Image synthesis is performed through a sequence of residual decoding blocks and progressive upsampling operations. Style modulation is implemented through adaptive instance normalization (AdaIN), where affine normalization parameters are generated directly from the style representation. This mechanism enables scanner-site appearance transfer while preserving anatomical structure encoded in the anatomical representation.

**Image Discriminator ($D^x$).** The image discriminator $D^x$ consists of a shared multi-scale 3D feature extractor jointly optimized through two output heads:

- the adversarial discriminator $D^x_a$, and
- the scanner-site predictor $D^x_p$.

The architecture is composed of three convolutional branches operating at progressively downsampled spatial resolutions. Each branch consists of stacked 3D convolutional layers equipped with squeeze-and-excitation attention modules, followed by global average pooling and a final projection layer producing $K + 1$ outputs, where $K$ corresponds to the number of scanner-site classes and the additional output is used for adversarial discrimination. The multi-scale formulation improves robustness to both local and global appearance variations.

**Anatomical Discriminator ($D^b$).** The latent anatomical discriminator $D^b$ consists of a shared latent feature extractor jointly optimized through two output heads:

- the latent adversarial discriminator $D^b_a$, and
- the latent scanner-site predictor $D^b_p$.

Unlike the image discriminator, $D^b$ operates directly on the anatomical latent representation. The network consists of successive 3D convolutional blocks with instance normalization and channel-attention modules, followed by adaptive global pooling and a final $1 \times 1 \times 1$ convolutional projection layer producing $K + 1$ outputs. This discriminator encourages invariance of the anatomical latent representation with respect to scanner- and acquisition-related appearance variations while preserving structural information.

All convolutions within the proposed framework are fully three-dimensional. This design enables the model to exploit volumetric contextual information across adjacent slices rather than processing 2D slices independently.

**Training Configuration and Computational Cost.** In all experiments, the noise dimension was set to $d_n = 8$, while $K$ corresponded to the number of scanner-site attributes. The loss weights were set as follows:

$$\lambda^x_{\text{adv}} = \lambda^b_{\text{adv}} = \lambda^b_{\text{pre}} = \lambda^{x,G}_{\text{pre}} = \lambda^{x,D}_{\text{pre}} = \lambda^x_{\text{lum}} = \lambda^x_{\text{struct}} = 1,$$
$$\lambda^x_{\text{rec}} = \lambda^s_{\text{cla}} = \lambda_{\text{lat}} = 10, \qquad \lambda^x_{\text{cc}} = 12.$$

In the combined reconstruction and cycle-consistency losses, SSIM and MAE were combined using a weighting factor $\lambda = 0.8$.

The model was trained for 100,000 iterations with batch size 1 on a single NVIDIA A100 80GB PCIe GPU, due to the memory requirements of fully 3D MRI volumes. Training the full model required approximately 40 GB of peak GPU memory and took about 100 hours. At inference time, the end-to-end harmonization runtime was evaluated on a reproducible subset of 100 3D MRI volumes, after 10 warm-up runs, using batch size 1 on the same GPU. Harmonization of a single MRI volume required $2.36 \pm 0.02$ s on average.

## 3 IMPLEMENTATION DETAILS OF THE BENCHMARK METHODS

This section provides additional details on the implementation of the image-level harmonization methods used as benchmarks in the main manuscript. For all benchmark methods, we followed the preprocessing and inference instructions provided by the original authors and used the model weights released with the corresponding implementations. This ensured that each benchmark was evaluated under the usage setting intended by its authors.

**STGAN** (Liu et al., 2023): STGAN was included as a style-transfer-based benchmark for MRI harmonization. The method assumes that an image can be represented through scanner-independent anatomical content and scanner-dependent style information. Harmonization is therefore performed by

preserving the anatomical content of the input image while transferring the image appearance toward that of a selected reference scan. Following the instructions provided with the original implementation, T1-weighted images were preprocessed using skull stripping (Ségonne et al., 2004), intensity nonuniformity correction with the N3 algorithm (Sled et al., 1998), and linear registration to the MNI standard space, using the ICBM 152 Nonlinear Symmetric atlas as reference (Fonov et al., 2009). Images were then resampled to an isotropic resolution of 1 $mm^3$. Since STGAN operates slice-wise, inference was performed according to the original implementation, using three consecutive slices with stride equal to one. As STGAN requires a reference image to define the target style, we used an image acquired on the Gyroscan Intera scanner as reference, consistently with the scanner selected as target domain in our comparison.

**IGUANe** (Roca et al., 2025): IGUANe was used as a generative adversarial benchmark for multi-scanner MRI harmonization. The method is based on a 3D extension of the CycleGAN framework and adopts a many-to-one harmonization strategy, in which images acquired from different scanners are mapped to a common reference domain. This formulation aims to reduce inter-scanner variability while preserving the three-dimensional anatomical structure of the input image. The preprocessing pipeline followed the instructions released with the original IGUANe implementation. Images were skull-stripped using HD-BET (Isensee et al., 2019), corrected for bias-field inhomogeneities using N4ITK (Tustison et al., 2010), and linearly registered to the MNI 1 $mm^3$ space using FSL-FLIRT (Jenkinson et al., 2002). After registration, the volumes were cropped to a spatial size of $160 \times 192 \times 160$ voxels. Intensity normalization was performed as specified by the authors, by normalizing brain intensities with respect to the median intensity within the brain mask while preserving the background at zero. Following the original many-to-one setting, IGUANe harmonizes images toward the SALD reference domain, which was selected by the authors because of its relatively large sample size and broad age coverage.

**DISARM** (Caldera et al., 2025): DISARM was included as an unsupervised 3D image-to-image translation benchmark for scanner-free MRI harmonization. We applied the scanner-free harmonization setting provided by the authors, using the released pretrained model and following the preprocessing and inference instructions of the original implementation. Accordingly, all input images were normalized to a standard orientation, corrected for bias-field inhomogeneities, and linearly registered to the MNI152 1 mm template (Jenkinson et al., 2002). Differently from STGAN, skull stripping was not applied, as it is not required by the DISARM preprocessing pipeline. In the scanner-free setting, the scanner-effect latent code is replaced by a random vector sampled from a standard Gaussian distribution, as specified in the original framework. The resulting scanner-free harmonized images were used as DISARM benchmark outputs in the comparative evaluation reported in the main manuscript.

## 4 VOXEL-INTENSITY HISTOGRAMS OF REPRESENTATIVE IMAGES

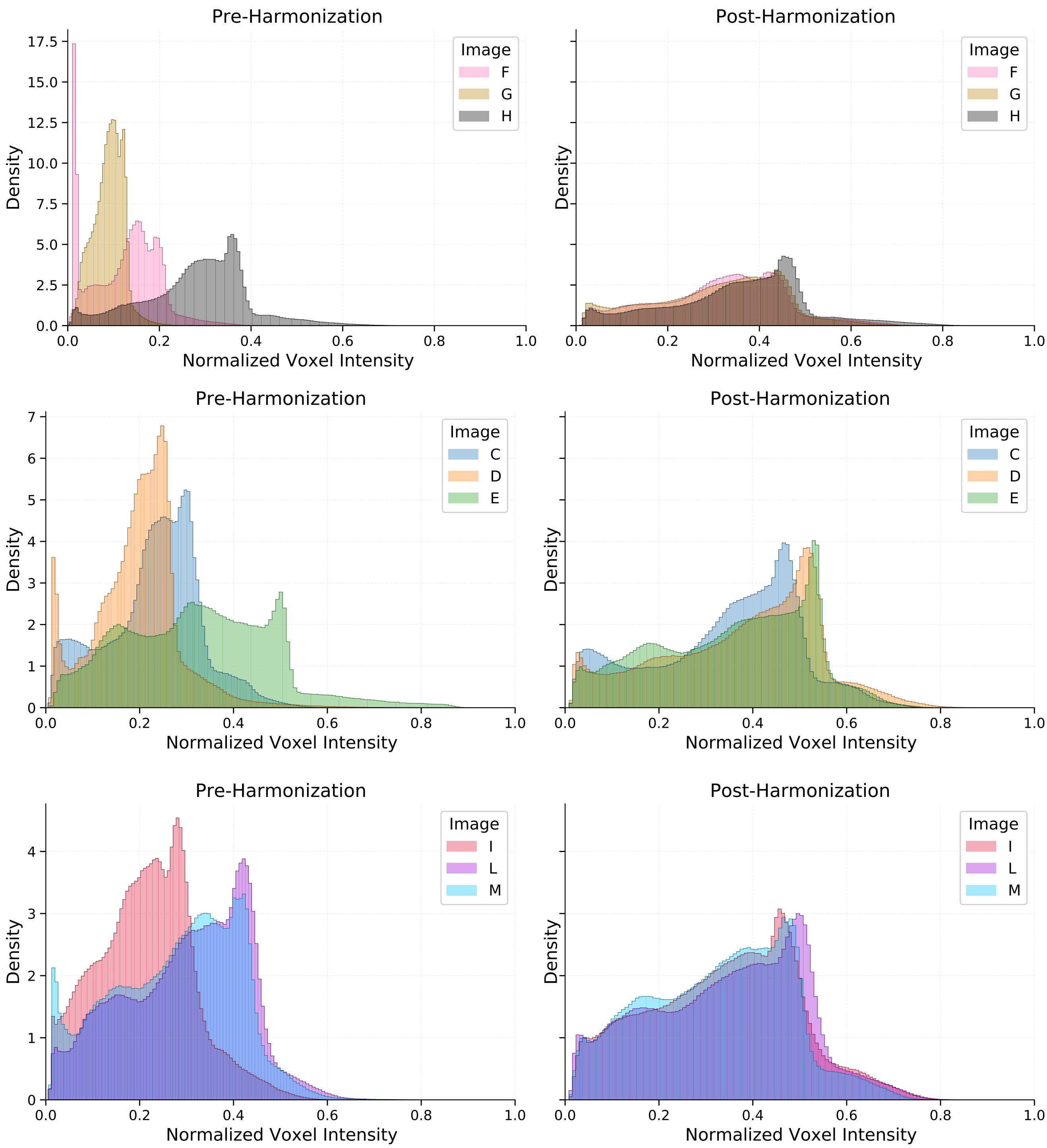


**Figure S1.** Voxel-intensity histograms of the representative images shown in Figure 3 of the main article before and after harmonization. The pre-harmonization panel shows the intensity distributions of the original images acquired from different scanner-site pairs, whereas the post-harmonization panel shows the corresponding harmonized images. A mild $3 \times 3 \times 3$ average pooling operation was applied only for visualization purposes before computing the histograms.

## REFERENCES

Caldera, L., Cavinato, L., Cappozzo, A., et al. (2025). Disarm: Disentangled scanner-free image generation via unsupervised image2image translation. In *International Workshop on Machine Learning in Clinical Neuroimaging* (Springer), 102–112

Fonov, V. S., Evans, A. C., McKinstry, R. C., Almli, C. R., and Collins, D. (2009). Unbiased nonlinear average age-appropriate brain templates from birth to adulthood. *NeuroImage* 47, S102

Isensee, F., Schell, M., Pflueger, I., Brugnara, G., Bonekamp, D., Neuberger, U., et al. (2019). Automated brain extraction of multisequence mri using artificial neural networks. *Human brain mapping* 40, 4952–4964

Jenkinson, M., Bannister, P., Brady, M., and Smith, S. (2002). Improved optimization for the robust and accurate linear registration and motion correction of brain images. *Neuroimage* 17, 825–841

Liu, M., Zhu, A. H., Maiti, P., et al. (2023). Style transfer generative adversarial networks to harmonize multisite mri to a single reference image to avoid overcorrection. *Human Brain Mapping* 44, 4875–4892

Roca, V., Kuchcinski, G., Pruvo, J.-P., et al. (2025). Iguane: A 3d generalizable cyclegan for multicenter harmonization of brain mr images. *Medical Image Analysis* 99, 103388

Ségonne, F., Dale, A. M., Busa, E., Glessner, M., Salat, D., Hahn, H. K., et al. (2004). A hybrid approach to the skull stripping problem in mri. *Neuroimage* 22, 1060–1075

Sled, J. G., Zijdenbos, A. P., and Evans, A. C. (1998). A nonparametric method for automatic correction of intensity nonuniformity in mri data. *IEEE transactions on medical imaging* 17, 87–97

Tustison, N. J., Avants, B. B., Cook, P. A., Zheng, Y., Egan, A., Yushkevich, P. A., et al. (2010). N4itk: improved n3 bias correction. *IEEE transactions on medical imaging* 29, 1310–1320
